# A Robust Deep Learning Approach for Position-Independent Smartphone-Based Human Activity Recognition

**DOI:** 10.3390/s18113726

**Published:** 2018-11-01

**Authors:** Bandar Almaslukh, Abdel Monim Artoli, Jalal Al-Muhtadi

**Affiliations:** Department of Computer Science, College of Computer and Information Sciences, King Saud University, Riyadh 11543, Saudi Arabia; aartoli@ksu.edu.sa (A.M.A.); jalal@ccis.edu.sa (J.A.-M.)

**Keywords:** human activity recognition, position detection, position-independent, deep learning, convolution neural networks, smartphone

## Abstract

Recently, modern smartphones equipped with a variety of embedded-sensors, such as accelerometers and gyroscopes, have been used as an alternative platform for human activity recognition (HAR), since they are cost-effective, unobtrusive and they facilitate real-time applications. However, the majority of the related works have proposed a position-dependent HAR, i.e., the target subject has to fix the smartphone in a pre-defined position. Few studies have tackled the problem of position-independent HAR. They have tackled the problem either using handcrafted features that are less influenced by the position of the smartphone or by building a position-aware HAR. The performance of these studies still needs more improvement to produce a reliable smartphone-based HAR. Thus, in this paper, we propose a deep convolution neural network model that provides a robust position-independent HAR system. We build and evaluate the performance of the proposed model using the RealWorld HAR public dataset. We find that our deep learning proposed model increases the overall performance compared to the state-of-the-art traditional machine learning method from 84% to 88% for position-independent HAR. In addition, the position detection performance of our model improves superiorly from 89% to 98%. Finally, the recognition time of the proposed model is evaluated in order to validate the applicability of the model for real-time applications.

## 1. Introduction

Over the last few years, the significant advancement in sensing technology has enabled researchers to develop intelligent systems that facilitate numerous applications. These applications are useful in our everyday activities, such as active and assisted living applications for smart homes [1,2,3,4,5], healthcare monitoring [6,7,8], and surveillance and security [9,10]. The aim of this paper is to develop an improved intelligent system which is able to recognize people’s activities of daily living (ADL), such as walking and jogging, in real-time. This system could be used to enhance the life quality of sick people by monitoring their activities. For example, elderly, disabled, or diabetic people are usually required to follow a well-defined procedure of daily exercises that is considered an important part of their treatment. Therefore, healthcare service providers can utilize the intelligent system to follow the behavior of their patients by recognizing different types of activities, such as walking, walking upstairs and lying. In addition, such intelligent systems may be used by healthy people as a proactive solution. For instance, a long-term analysis of healthy people’s activities could help as an early detection for some diseases [11] or as a way to improve their health by informing them of their progress in daily activities. In this study, we focus on developing an intelligent system that predicts the activity of a person effectively and in real-time with the aid of an attached accelerometer that is available on a body-attached smartphone.

In order to develop the aforementioned intelligent system, we need a way to identify the human activities. The research field that is steered towards identifying these activities is called human activity recognition (HAR). An HAR system can be developed by integrating sensing technology and machine learning approaches. In this study, we focus on the machine learning part to develop an effective and efficient HAR system.

Recently, modern smartphones equipped with a variety of embedded-sensors, such as accelerometers and gyroscopes, have been employed as an alternative platform for human activity recognition (HAR). The smartphone-based HAR system is a machine learning model that is deployed on the subject’s smartphone and continuously recognizes her/his activities while the smartphone is attached to a part of the person’s body. This system takes the advantages of the current smartphone computing resources to develop a real-time system.

Generally, developing such a system is performed in four fundamental steps: data collection, windowing, feature extraction and classification. Feature extraction is considered to be the main critical step, since it determines the overall performance of the model. This step can be accomplished either using traditional machine learning methods or deep learning approaches. In traditional machine learning methods, the domain experts manually extract heuristic or handcrafted features in both the time and frequency domains. There are many time domain features such as max, min, mean, correlation, standard deviation, etc. Also, there is a variety of frequency domain features such as energy, entropy, time between peaks, etc. However, handcrafted features have some limitations in both domains. First, they depend on domain knowledge and human experience. This knowledge could aid in a certain problem with specific settings, but it cannot be generalized for the same problem with different settings. In addition, the human experience is only used to extract shallow features, such as statistical information, but fails to discriminate between activities with approximately similar pattern s (such as sitting and standing activities in HAR). There are many works that have used the traditional machine learning approaches to build smartphone-based HAR [12,13]. To overcome the above limitation, deep learning approaches are used. In deep learning approaches, the features can be learned automatically using more than one hidden layer instead of being manually extracted by the domain expert. The deep architecture of these approaches enables the extraction of high-level representation (deep features) which are more suitable for complex problems such as HAR. Recently, deep learning approaches have been employed to build a robust smartphone-based HAR [14,15].

To date, the majority of smartphone-based HAR systems require the carried smartphone to have a fixed on-body position [13,16,17]. In the literature, these systems are called position-dependent HARs. However, in real-life applications, people usually carry their smartphones in different locations, which reduces the accuracy of the recognition, as sensor readings (motion information) can be very dissimilar for the same activity in different on-body positions. Thus, developing a position-independence or position-aware solution is the main challenge for smartphone-based HAR systems [18]. In the this paper, we fundamentally focus on developing a position-independent HAR using deep learning approaches. In the deep learning context, the model is trained in an end-to-end manner; thus the need for manual handcrafted feature extraction is eliminated.

This paper is considered an extension to our previous paper [14]. In that paper, we proposed a deep learning architecture using the Convolutional Neural Network (CNN) together with time-domain statistical features that effectively represented the raw time series data of smartphone-based HAR. In that paper, we mainly focused on position-dependent HAR, i.e., the target user had to fix the sensor in a specific position. In the current paper, as an extension, without losing generality, we alter the proposed CNN architecture to produce an effective position-independent HAR. In addition, we show that the altered model is able to produce an effective position detection model which is the first step towards a building position-aware HAR. Moreover, the effectiveness of the new model against different body positions (chest, forearm, head, shin, thigh, upperarm, and waist) is demonstrated.

The main contribution of this paper are as follows:

- We propose a deep learning architecture that uses CNN together with time-domain statistical features to produce an effective position-independent HAR.
- We also show that the proposed model produces a robust on-body position detection system.
- We perform comprehensive experiments to prove the robustness of the proposed model in solving the position-independent HAR and position detection problem. In addition, we compare the results with the state-of-the-art method in [19] using a variety of real world settings.

The reminder of the paper is organized as follows. In Section 2, we survey the related studies. The materials and methods of the proposed model are explained in Section 3. Section 4 presents the results for the proposed method with RealWord HAR dataset. To show the applicability of the proposed model for real-time application, we assess the recognition time of the proposed model in Section 5. Finally, the conclusions and future works are given in Section 6.

## 2. Related Works

The majority of the proposed methods ask the user who is joined to the data collection phase, to place the smartphone in a specified position, such as the thigh pocket or other positions. This setting restricts the manner in which users carry and use their smartphone while performing daily activities. To reduce this problem, researchers have proposed a position-independent and position-aware HAR where the user is free to attach the smartphone to any on-body position [18,20,21,22,23,24,25,26].

In the literature, two main methods have been used to develop an HAR model that enables the user to freely attach the phone to any on-body position: position-independent and position-aware HAR. In the first approach, the model is trained using data from mixed sensors obtained from different positions. In addition, special handcrafted features are used to mitigate the variation in motion data of the same activity in different positions [20,21,22,23]. The limitation of this approach is attributed to the handcrafted features extracted by the domain experts. These features are shallow and cannot be generalized for the same problem with different settings. The second approach is based on building two or more classifier levels: the first-level classifier recognizes the specific position of the sensor and the second one is used to recognize the activity for this specific position [18,24,25,26]. This approach is called position-aware HAR. The limitation of this method is that it is highly computational expensive to run on resource-limited devices (smartphone) since we need to recognize the position of the sensor first and then the activity for each instance. In addition, we need to train a specific model for each position.

Deep learning approaches have intensively been used in different studies to produce an effective smartphone-based HAR. However, there are several deep learning models available in the literature, including convolutional neural networks (CNNs) [27], stacked autoencoders (SAEs) [28], deep belief networks (DBNs) [29], and long short-term memory networks (LSTMs) [30]. Therefore, we categorize these studies into five classes based on the deep learning model used in Table 1. All of these works use deep learning models to produce position-specific (position-dependent) smartphone-based HAR. We claim that this work is the first to target position-independent smartphone-based HAR using deep learning.

In this study, we demonstrate that deep learning approaches noticeably outperform the traditional machine learning approaches concerning the position-independent HAR. In addition, we use a single deep learning classifier that outperforms the position-aware (multi-level classifier) approach considerably. Finally, we report a significant improvement in the accuracy of position detection over the state-of-the-art solution [19].

## 3. Materials and Methods

This section is three-fold. In Section 3.1, we recall the general architecture of the CNN model. After that, a description of the used dataset is given in Section 3.2. Finally, the proposed position-independent CNN architecture is illustrated in Section 3.3.

### 3.1. Convolutional Neural Network (CNN)

The convolutional neural network (CNN) [27] is a special multi-layer neural network (NN) structure [43]. The architecture of CNN consists of two main parts: several convolution/sampling layers, and a fully connected network. The convolution and sampling layers work as a feature extractor. At the topmost part, a fully-connected network is added to learn classification weights. However, CNNs mainly consist of three main layers: convolutional, pooling (down-sampling), and fully-connected. Subsequently, the description and intuition behind these layers are given. The convolutional layer consists of a set of filters. These filters target the extraction of local features (feature maps) from the input data (image or snesor). Each feature map is calculated using one filter. The feature maps are generated by sliding a corresponding filter over the input and computing the dot product (convolution operation). Each neuron of the feature maps only connect to a small region of the input called the receptive field which is equal to the filter size. All neurons of a single feature map share the same weights (filters). The advantage of sharing weights is the reduced number of parameters (weights) which makes the computation more efficient. In addition, this provides the ability to detect a specific pattern, irrespective of its location in the inputs. The size of the generated feature map basically depends on the stride number and filter size.

The pooling layer breaks the previous feature maps into disjointed regions to be a size of (R×R); then one output from each region is determined. The output of each region is either the maximum or average of all values [44]. Even though the pooling layer reduces the resolution of the feature maps, it can produce much fewer features that are invariant with a small transformation and distortion. Another advantage of the pooling layer is that is reduces the dimensionality of features which makes the computation more efficient.

The top layers of CNNs are one or more **fully-connected** layers. These layer/s aim to represent the global features of the input data and learn the classification parameters. The topmost layer is a softmax classifier, which aims to predict the posterior probability for each class label [45].

### 3.2. Dataset

In this study, we used a publicity available dataset (RealWorld HAR) [25] to build and evaluate the proposed method. The dataset was collected from a group of 15 volunteers. Each person was carrying a set of smartphones (Samsung Galaxy S4) and a smart-watch (LG G Watch R) located at seven different on-body positions (chest (P1),forearm (P2), head (P3), shin (P4), thigh (P5), upper arm (P6), and waist (P7)). The performed activities by each person were climbing downstairs (A1), climbing upstairs (A2), jumping (A3), lying (A4), standing (A5), sitting (A6), running/jogging (A7), and walking (A8). For each activity, the acceleration data of all on-body positions were collected concurrently at a sampling rate of 50 Hz.

The advantages of this dataset over other available datasets are as follows:

1. The data was collected in realistic settings. For example, volunteers walked through the city or jogged in a forest. In addition, they were not instructed to perform the activities in a specific way (it was up to them), e.g., they could walk at any speed or they could sit/stand while eating, drinking or using the phone.
2. This dataset considered all related body positions in the context of common daily activities.
3. The data was collected from seven females and eight males with different physical characteristics (age 31.9 ± 12.4, height 173.1 ± 6.9, and weight 74.1 ± 13.8).
4. The dataset has a large number of instances, which makes it an appropriate resource to build deep learning models. When we used a one-second window length that overlapped by half, the distribution of the data was as listed in Table 2 and Table 3 for each on-body position and each activity, respectively.

### 3.3. The Proposed Position-Independent Model

**Figure 1:**
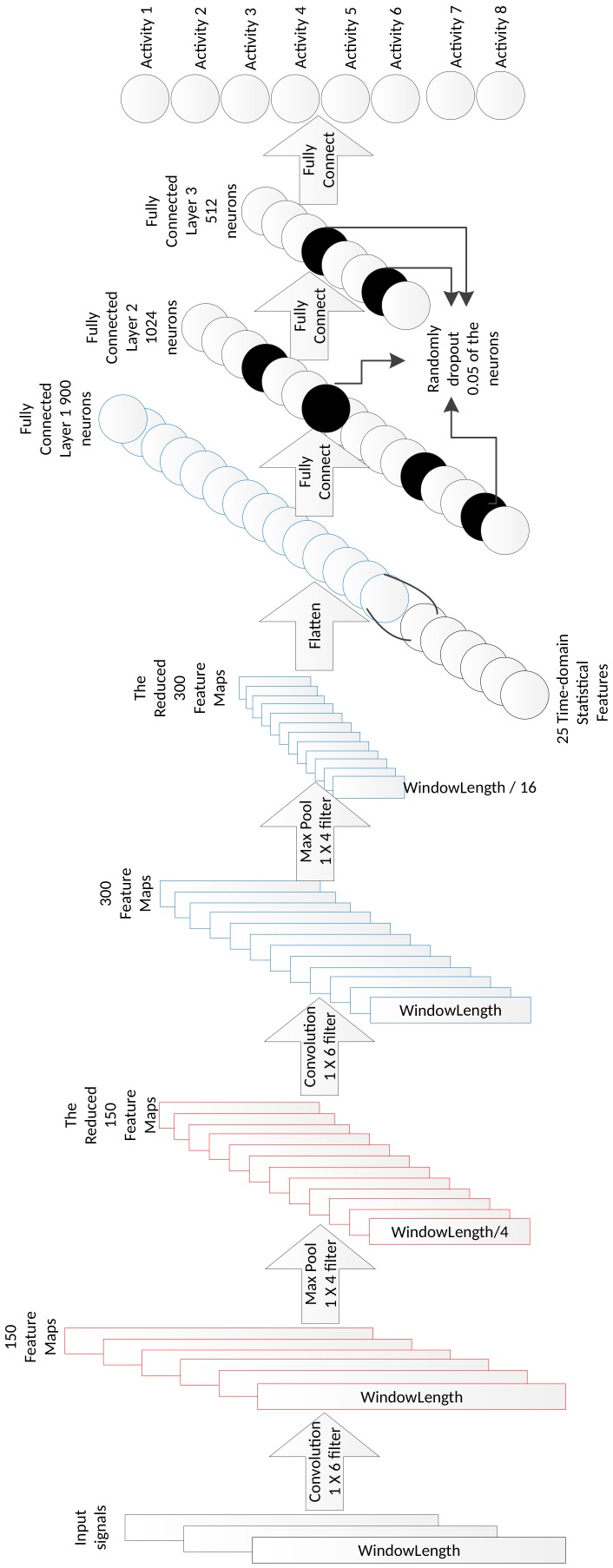
The architecture and hyper-parameters of the proposed model.

The CNN architecture of the proposed model is illustrated in Figure 1. The input channels of the model are windows of raw accelerometer data over three axes. These windows are pre-processed using mean-centering to transform the raw data into a form that is suitable for learning the optimal weights without any bias.

The first convolution layer learns 150 convolution filters that are used to extract a detailed representation from the input data. Then, the dimensionality of the first convolution layer is reduced four-fold using the max-pooling layer of size 1×4. After that, the second convolution layer learns 300 filters that are used to transform the reduced feature maps of the first convolution layer to a more abstract representation. Also, the second convolution layer is reduced by four-fold using a max-pooling layer. The size of the filters used in both convolution layers are 1×6. In addition, the ReLU activation function is also used in both convolution layers. The advantages of using the ReLU activation function are tha it experimentally produces a better performance and is computationally efficient compared to other types of activation functions.

The abovementioned layers are used to extract the features. Then, the classification weights need to be learnt as follows. The reduced representation of the second max-pooling layer is flattened to be a 1D vector. The flattened vector is stretched by concatenating the time-domain statistical features, as in [14]. Then, the stretched vector is connected to a fully-connected layer made up of 1024 neurons which is connected to another fully-connected layer made up of 512 neurons. Finally, the topmost is a soft-max (output) layer which is applied to calculate the posterior probability over the activity labels.

To learn the model wights, the training data is fed into the network, and then the network weights are optimized using a modified version of the stochastic gradient descent (Adam) and backpropagation algorithm.

### 3.4. Model Parameters Selection

In the context of deep learning, it is known that the model is learned from the data directly in an end-to-end manner, which means a labor-intensive hand-crafted features extraction is not required by the human (domain experts). Unfortunately, intensive human efforts are needed in deep learning for the model selection process. This process is conducted by finding the hyper-parameter values that achieve the best performance. Generally, there are three different approaches to finding the best hyper-parameter values: manually, based on prior experiences; randomly from a given space of candidate hyper-parameter values; and exhaustive grid search.In this paper, we use an efficient grid search which initially uses coarse ranges of the hyper-parameter values, then we use a narrow range. In addition, we use our experiences in deep learning and previous work [14] to choose these ranges.

The proposed CNN architecture has many hyper-parameters. These hyper-parameters should be chosen carefully since they control the performance of the model. The grid search technique was used to figure out the optimal combination of these parameters. In this paper, we mainly tuned the following hyper-parameters: optimization algorithm, learning rate, conventional filter size, pooling size, dropout rate, the number of filters (feature maps) in each convolutional layer, the number of convolution layers, and the number of full-connected layers. To select the best hyper-parameters of the proposed model, we used the data from subject 1 in the dataset as a validation set.

**Optimization algorithm**: A traditional stochastic gradient descent (SGD) method is used to tune network weights iteratively, based on training data. There have recently been many variations of gradient descent optimization algorithms, such as Insofar, RMSprop, Adadelta, and Adam (see [46]). After testing all of these algorithms to train the proposed model, we adopted the Adam as it revealed slightly higher performance

**Learning rate**: This is considered to be the most critical hyper-parameter, since it can change the accuracy of the model significantly. Generally, a small learning rate takes a very long time to converge, whereas a large learning rate may achieve a good performance initially, but not converge as a result of overshoot. However, in the original paper of the Adam algorithm [47], it was recommended to use 0.001 as an initial value for the learning rate. We selected a learning rate of 0.0003, which reported the highest performance within a search range between (0.00001 and 0.2).

**Figure 2:**
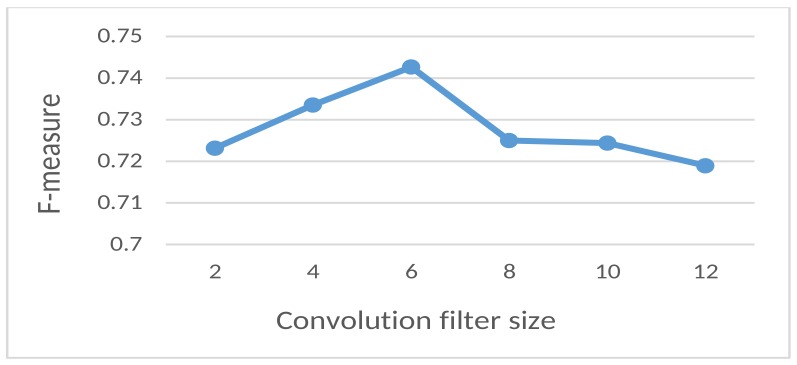
Dependency between the convolution filter size and the F-measure.

**Conventional filter size**: In Figure 2 the dependency between convolution filter size and F-measure of the proposed model is shown. The best performance was reached when the filter size was six.

**Figure 3:**
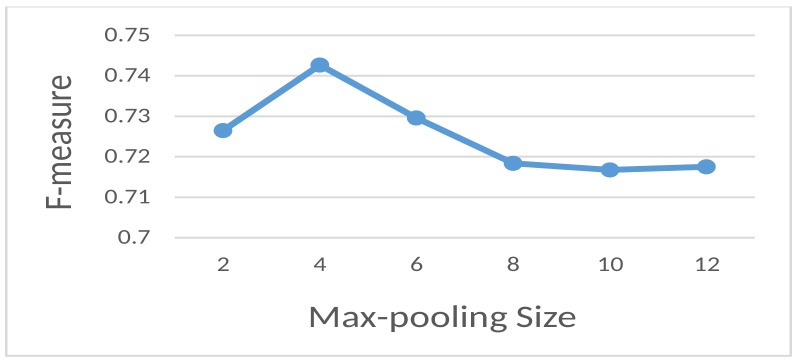
Dependency between the pooling size and the F-measure.

**Pooling size**: In Figure 3, we show the relation between the pooling size and the F-measure of the proposed model. The maximum value was found when the max-pooling size was equal to four.

**Figure 4:**
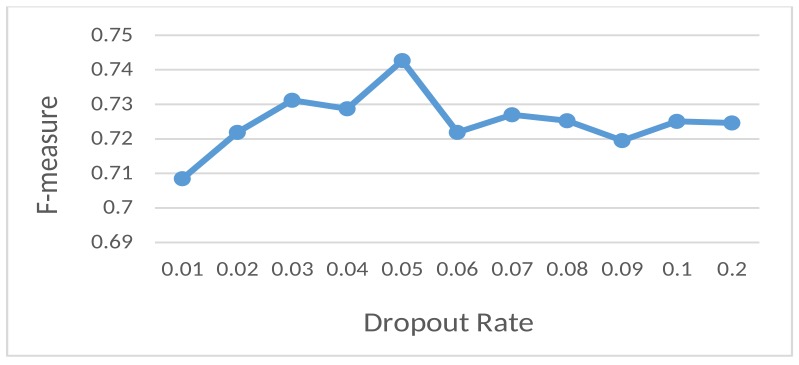
Dependency between the F-measure and the dropout rate in the fully-connected layers.

**Dropout rate**: The influences of different dropout rates on the F-measure of the proposed model are shown in Figure 4. We noticed that the F-measure immediately started decreasing when the dropout rate was greater than 0.1, so we only investigated rates between 0.0 and 0.1. We achieved the best F-measure when the dropout rate was 0.05.

**Figure 5:**
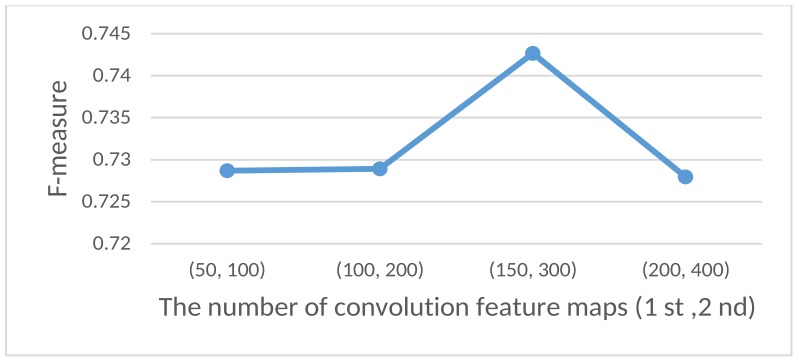
Dependency between the F-measure and the number of feature maps in the first and second convolution layers and.

**The number of convolution feature maps**: The number of feature maps (filters) in both convolution layers were continuously changing. The dependency between the number of feature maps and the F-measure of the model is shown in Figure 5.

**The number of fully-connected layers**: The optimal number of fully-connected layers was determined to be two layers after spanning a range of 0 to 4 layers. The procedure we used to add a new fully-connected layer is described as follows. First we use one fully-connected layer with many neurons. If it does not work, we use two layers with a few neurons in each layer, then finally by combining these techniques (two fully-connected layers with many neurons in each layer) and continuing in the same fashion for layers three and four. For the number of neurons in each layer, we used values ranging from 256 to 2048 neurons, by adding 50 in each trial.

**The number of convolution layers**: Initially, we used a single convolution layer. Then, we used two convolution layers, which increased the accuracy significantly, but more layers did not increase the accuracy significantly. It is obvious that the computational cost increases as the number of convolution layers increases. We applied the same procedure to add a new fully-connected layer with feature maps in the range from 50 to 400 feature maps, by adding 50 in each trial.

## 4. Experiments

In this section, we evaluate the performance of the proposed model and compare it with the state-of-the-art solution [19]. In that work [19], a position-aware HAR was proposed based on a three level classifier: the first-level classifier was used to distinguish between dynamic and static activities, the second-level classifier was used to detect the position of the sensor, and the third-level had a set of activity recognition classifiers each belonging to specific position. This method uses handcrafted features and Random Forest classifier [48].

However, this section is three-fold. First, we give the experimental settings and the evaluation approaches in Section 4.1. In Section 4.2, we evaluate the performance of proposed model concerning activity recognition using different scenarios that are usually used in the literature as listed in Table 4. Moreover, the performance of the proposed model concerning position detection is given in Section 4.3.

### 4.1. Experimental Settings

To make the comparison more realistic, we used the same settings as those used by [19]. We only used an acceleration sensor as it consumes a low level of power which enables continuous sensing over time and has a long battery life [13,19]. In addition, it has has been proven that acceleration sensors are appropriate for activity/position recognition [13,49,50]. The second important setting is the window length for which we used windows of one-second length and overlap by half, as done in [19].

It is worth mentioning that the performance results shown in the following sections were calculated by the aggregating results of all subjects. For the subject-independent model, we used the leave-one-subject-out (L1O) approach which trains a single model for each subject. This model was trained using all subjects’ labeled data, except the target subject who was used to evaluate the performance of the model. For the subject-dependent model, we trained a single model for each subject. This model was trained and evaluated using the labeled data from the same target subject. To evaluate the subject-dependent model, we used 10-fold cross validation with stratified sampling in order to guarantee that all folds had the same ratio of classes.

### 4.2. Activity Recognition

In this section, three cases are investigated. In Section 4.2.1 and Section 4.2.2 position-independent HAR results are presented for subject-dependent and subject-independent cases. Moreover, we present the position-dependent HAR results for subject-independent in Section 4.2.3.

#### 4.2.1. Position-Independent and Subject-Dependent HAR

The performance of the proposed model in this scenario is illustrated in Table 5. The table shows that the correct activity was recognized with an F-measure of 88%. Referring to the previous state-of-the-art study in [19], we found that that the average F-measure of our model outperformed that in Reference [19] by 8% and 4% for the position-independent and position-aware HAR, respectively.

**Figure 6:**
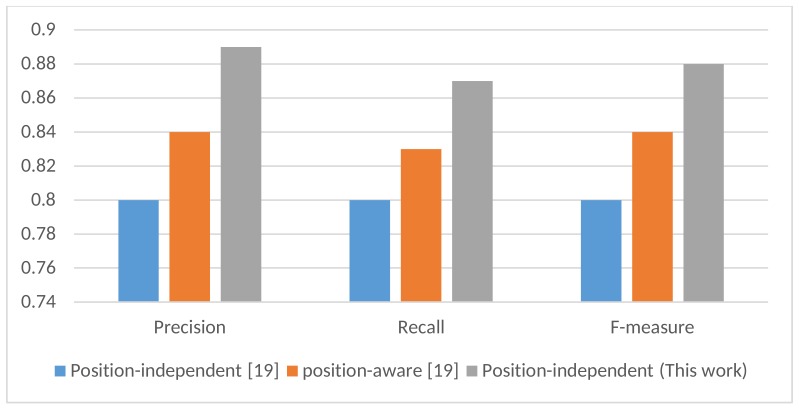
F-measure, precision, and recall of the proposed model compared to the position-independent and position-aware HAR in [19].

The position-aware HAR proposed in [19] is computationally expensive, since for each instance, three classifiers need to be applied. In addition, a separate classifier needs to be built for each position. However, it is clear from Figure 6 that the F-measure, precision, and recall of the proposed model all outperformed the position-aware HAR significantly in [19]. We can conclude that the proposed deep learning model better captures features that are less sensitive to the variation in motion data of the same activity at different positions.

**Figure 7:**
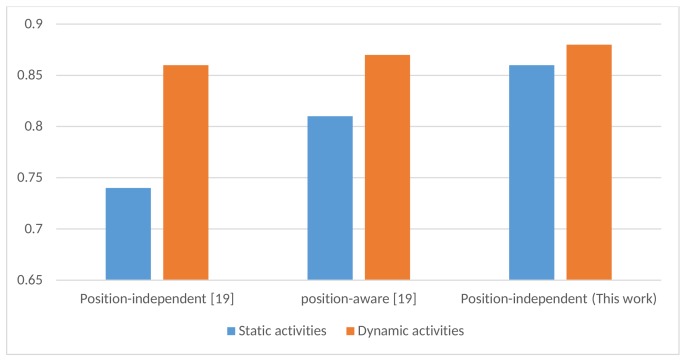
F-measures for the static and dynamic activities of the proposed model compared to the position-independent and position-aware HAR in [19].

In HAR literature, it is challenging to distinguish between static activities, such as lying, standing, and sitting, since the human body only has slight acceleration in these activities. This problem was addressed in [19] by classifying the activity as static or dynamic, and then considering specific features for the static activities. In this work, we let the proposed deep learning model implicitly distinguish between the static activities without adding any new classifier. In Figure 7, we show how the proposed method improved the performance of static activities significantly as well as a slight improvement in the performance of the dynamic activities. More precisely, the performance increased by 5% and 1% for static and dynamic activities, respectively.

#### 4.2.2. Position-Independent and Subject-Independent HAR

The activity recognition scenario presented in this section is plug-and-play. This means that the target user does not need to label any data and can freely carry the smartphone on any of the seven on-body positions. The results of the plug-and-play mode for the proposed model are shown in Table 6. The table shows that the correct activity was recognized with an F-measure of 78%. We noticed that the performance of the proposed model in the plug-and-play scenario was similar to the best position-dependent (waist) in [19] (see next section). It is worth mentioning that the model in [19] does not evaluate the performance of the plug-and-play scenario.

#### 4.2.3. Position-Dependent and Subject-Independent HAR

In this scenario, we evaluated the proposed method against different on-body positions. It was important to evaluate the position-dependent HAR since it could be used as a final step for the position-aware HAR. Table 7 shows the F-measure of the proposed model across all seven positions.

**Figure 8:**
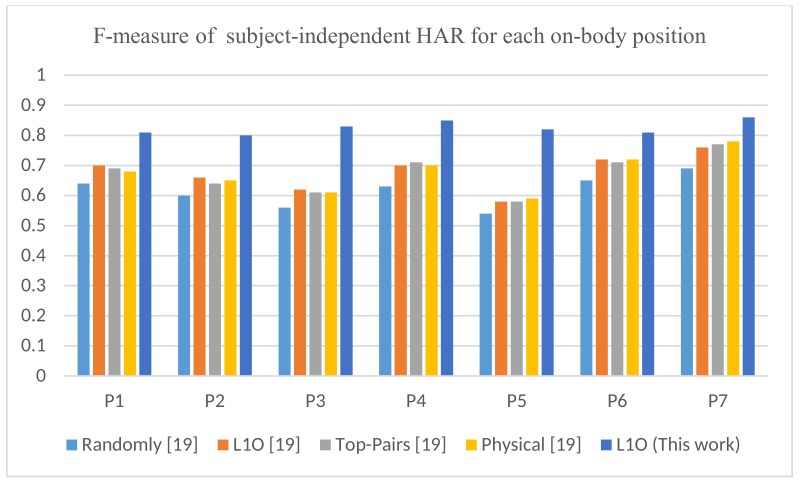
F-measure of position-dependent and subject-independent HAR for this work using L1O compared to the work in [19] with different evaluation approaches.

In [19], five different approaches were presented which used to evaluate the performance of the subject-independent model. The descriptions of these approaches were given in [19]. However, these approaches achieved a comparable performance, as shown in Figure 8. It was concluded that top-pairs and physical approaches for the waist (P7) position performed slightly better while they processed significantly less data. In our opinion, the physical and top-pair approaches are not practically feasible for real-life applications since it is not always guaranteed that there will be a labeled data of subjects that have approximately similar physical characteristics as the target subject. However, it is obvious in Table 7, that the proposed method significantly surpasses the performance in [19] across all positions.

In addition, the previous study in [19] showed that the leave-one-subject-out (L1O) approach cannot scale for a large number of subjects. This is caused by the fact that the classifier tends to learn the dominated behavior across all subjects and loses the behavior of individual subjects. In this paper, we prove the opposite trend, since in a deep learning context, the model performance increases with a larger amount of data. To prove that, we show that the proposed model that use leave-one-subject-out approach significantly outperforms the method in [19] (see Figure 8).

### 4.3. Position Detection

In this section, the strength of the proposed model in position detection is evaluated. In the deep learning context, it is common to use the structure or weights of one problem to solve other very different problems such as face detection and object recognition. This is not the case in traditional machine learning approaches that use handcrafted features that could aid in a certain problem with a specific setting, but it cannot be generalized for the same problem with different settings. This explains why we use the proposed HAR architecture for position detection, revealing excellent performance. However, independent of the performed activity, we evaluated the performance of position detection in two different scenarios: subject-dependent (Section 4.3.1) and subject-independent HAR (Section 4.3.2). We evaluated the performance of the position detection as it has been proven that the accuracy of activity recognition can be improved significantly if the on-body position is known [25]. More precisely, in position-aware HAR, the first step is to detect the position of the sensor, followed by recognition of the activity using the classier of the corresponding position. This means that the performance of the activity recognition is led by the accuracy of the position detection model. In addition, the information of smartphone placement is considered to be an important source for context-aware applications [51,52,53].

#### 4.3.1. Subject-Dependent Position Detection

The position detection performance of the proposed model is shown in Table 8. The table shows that the correct position was detected with an F-measure of 98%. Recall the state-of-the-art study in [13] which reported an average F-measure of position detection of 89%.

In [19], the authors applied a two-level classifier for activity-aware position detection: the first one was used to distinguish between dynamic and static activities and the second one was used to detect the position. On the other hand, the proposed method uses only a single classifier to detect the sensor’s position. However, even though we used a single classifier, the performance of our model surpassed the method in [19] significantly. More precisely, our proposed model outperformed the method presented in [19] by 10% on average F-measure.

#### 4.3.2. Subject-Independent Position Detection

**Figure 9:**
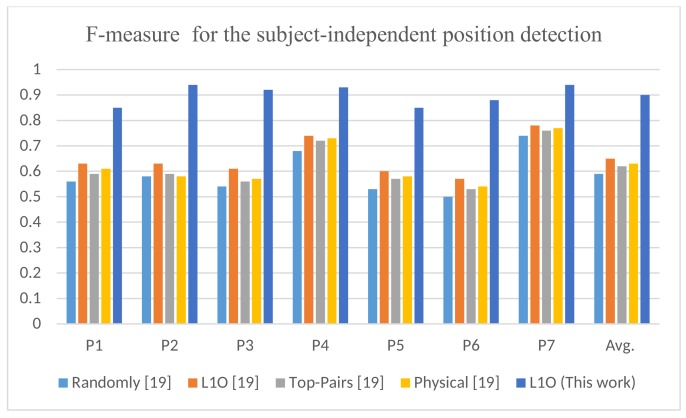
F-measure of subject-independent position detection for this work compared to the work in [19] with different evaluation approaches.

The position detection performance of the proposed model is shown in Table 9. The table shows that the correct position was detected with an F-measure of 90%. Five different approaches were given in [19].The descriptions of these approaches are given in [19]. These approaches achieved a very low accuracy range (59–65%) which made the authors in [19] to suggest further investigations. In this paper, we partially resolved this issue using the deep learning approach which improved the average position detection by 25%, as shown in Figure 9.

## 5. Recognition Time

We implemented the proposed model using a Python library named Tensorflow, which is available for smartphones. Then, we deployed the optimized model that learned in the training phase on a Nexus 5X Android smartphone to measure the recognition time. The advantage of this measure is its ability to reflect the applicability of the model for online (real-time) smartphone-based HAR applications. As mentioned in [15], it is acceptable for online (real-time) applications to predict 1–5 activities per second. Therefore, in the test (prediction) phase, we measured how many activities we could recognize per second using the proposed model that was deployed on a smartphone. We found that the proposed model could recognize approximately 40 activities/second using a smartphone (Nexus 5X Android). This means that the proposed model is appropriate for real-time applications.

It worth mentioning that the learning of a deep model usually spans for long time (i.e., several hours or a few days) and requires high computational resources. However, this is not an issue in the present study, since the learning phase was conducted off-line on powerful computers (with many GPUs).

## 6. Conclusions and Future Work

In this paper, we proposed a deep learning architecture that uses CNN together with time-domain statistical features and demonstrated its capability to produce an effective position-independent HAR. In addition, we applied the proposed architecture to the position detection problem. We noticed that the proposed model revealed excellent results compared to the state-of-the-art method presented in [19]. These results support claims that deep learning approaches are more generic and robust than other traditional machine learning methods.

For position-independent and subject-dependent HAR, the proposed method revealed good results compared to the state-of-the-art method. More precisely, we used position-independent HAR with a single classifier which considerably outperformed the state-of-the-art position-aware method by 4%. In addition, we evaluated the performance of our model in the plug-and-play (position-independent and subject-independent) scenario which has rarely been evaluated in the literature.

For position detection, the proposed model revealed a very high performance with an F-measure of 98% in subject-dependent mode. Moreover, the proposed model superiorly outperformed the state-of-the-art study by 10% in subject-dependent mode. In the subject-independent position detection scenario, we revealed a very good performance (F-measure = 90%) compared to the state-of-the-art method that struggled to solve this scenario effectively.

We believe that the problem of position-independent HAR needs further investigation to develop a very accurate model. Therefore, in the next works, we will investigate different approaches to enhance the accuracy. First, we will implement position-aware HAR based on deep learning approaches. Furthermore, we will augment the fully-connected layer with position-independent handcrafted features instead of the statistical features that were used in this study.

## Figures and Tables

**Table 1:** Classification of related deep learning methods for smartphone-based human activity recognition (HAR). CNN: convolutional neural network; DBN: deep belief network; LSTM: long short-term memory network; SAE: stacked autoencoder.

Model Type	Ref.
CNN	[15,31,32,33,34,35]
SAE	[36,37]
DBN	[38]
LSTM	[39,40]
Hybrid approaches	CNN+LSTM in [41] and DBN+HMM in [42]

**Table 2:** Data distribution over the on-body positions deduced from the RealWorld HAR dataset [25].

Position	Number of Instances
$P_{1}$	135,288
$P_{2}$	132,757
$P_{3}$	135,248
$P_{4}$	135,248
$P_{5}$	135,109
$P_{6}$	135,286
$P_{7}$	135,348
**Total**	**944,356**

**Table 3:** Data distribution over the activities deduced from RealWorld HAR [25].

Activity	Number of Instances
$A_{1}$	105,529
$A_{2}$	130,834
$A_{3}$	20,130
$A_{4}$	134,749
$A_{5}$	132,923
$A_{6}$	134,469
$A_{7}$	150,015
$A_{8}$	135,707
**Total**	**944,356**

**Table 4:** List of evaluation scenarios often used in the literature.

Approach	Attributes
Position-dependent	1- The target user has to fix the smartphone to a specific on-body position.
	2- Training and test data are collected from specific on-body positions.
Position-independent	1- The target user is free to attach the smartphone to any on-body position.
	2- Training and test data are collected from different on-body positions.
	3- A single classifier with position-independent features is used.
Position-aware	1- The target user is free to attach the smartphone to any on-body position.
	2- Training and test data are collected from different on-body positions.
	3- Tow-level classifiers are used, the first to detect the position and the second level
	to recognize the activity using a corresponding position-dependent classifier.
Subject-dependent	1- The target user has to collect and label data.
	2- Training and test data belong to the same subject.
Subject-independent	1- The target user only uses the already labeled data from other people.
	2- Training and test data are from a disjointed set of subjects.

**Table 5:** Precision, Recall, and F-measure of the proposed model for position-independent and subject-dependent HAR.

Class	Precision	Recall	F-Measure
$A_{1}$	0.86	0.84	0.85
$A_{2}$	0.86	0.83	0.85
$A_{3}$	0.98	0.91	0.94
$A_{4}$	0.95	0.93	0.94
$A_{5}$	0.77	0.86	0.81
$A_{6}$	0.84	0.83	0.83
$A_{7}$	0.95	0.90	0.92
$A_{8}$	0.88	0.89	0.88
**avg.**	**0.89**	**0.87**	**0.88**

**Table 6:** Precision, Recall, and F-measure of the proposed model for position-independent and subject-independent HAR.

Class	Precision	Recall	F-Measure
$A_{1}$	0.77	0.77	0.76
$A_{2}$	0.78	0.74	0.76
$A_{3}$	0.91	0.88	0.89
$A_{4}$	0.87	0.81	0.84
$A_{5}$	0.61	0.82	0.69
$A_{6}$	0.65	0.62	0.62
$A_{7}$	0.98	0.84	0.89
$A_{8}$	0.76	0.76	0.75
**avg.**	**0.79**	**0.78**	**0.78**

**Table 7:** F-measure of position-dependent and subject-independent HAR for this work over all seven positions.

Position	F-Measure
$P_{1}$	**0.81**
$P_{2}$	**0.80**
$P_{3}$	**0.83**
$P_{4}$	**0.85**
$P_{5}$	**0.82**
$P_{6}$	**0.81**
$P_{7}$	**0.86**

**Table 8:** Precision, Recall, and F-measure of proposed model for subject-dependent position detection.

Class	Precision	Recall	F-Measure
$P_{1}$	0.96	0.97	0.97
$P_{2}$	0.98	0.98	0.98
$P_{3}$	0.98	0.97	0.98
$P_{4}$	0.99	0.97	0.98
$P_{5}$	0.97	0.96	0.97
$P_{6}$	0.97	0.97	0.97
$P_{7}$	0.98	0.99	0.99
**avg.**	**0.98**	**0.97**	**0.98**

**Table 9:** Precision, Recall, and F-measure of the proposed model for subject-independent position detection.

Class	Precision	Recall	F-Measure
$P_{1}$	0.87	0.85	0.85
$P_{2}$	0.91	0.97	0.94
$P_{3}$	0.93	0.91	0.92
$P_{4}$	0.94	0.93	0.94
$P_{5}$	0.86	0.86	0.85
$P_{6}$	0.89	0.88	0.88
$P_{7}$	0.94	0.94	0.94
**avg.**	**0.91**	**0.91**	**0.90**

## Abbreviations

The following abbreviations are used in this manuscript:

HAR	Human Activity Recognition
CNN	Convolutional Neural Network
L1O	Leave-One-Subject-Out
